# In Vitro Selection of Cell-Internalizing DNA Aptamers in a Model System of Inflammatory Kidney Disease

**DOI:** 10.1016/j.omtn.2017.06.018

**Published:** 2017-06-27

**Authors:** Glory Ranches, Melanie Lukasser, Herbert Schramek, Andreas Ploner, Taras Stasyk, Gert Mayer, Günter Mayer, Alexander Hüttenhofer

**Affiliations:** 1 Division of Genomics and RNomics, Biocenter, Medical University Innsbruck, Innsbruck 6020, Austria; 2 Division of Medical Biochemistry, Biocenter, Medical University Innsbruck, Innsbruck 6020, Austria; 3 Division of Nephrology and Hypertension, Department of Internal Medicine IV, Medical University Innsbruck, Innsbruck 6020, Austria; 4 Sandoz GmbH, Biochemiestrasse 10, Kundl 6250, Austria; 5 Division of Cell Biology, Biocenter, Medical University Innsbruck, Innsbruck 6020, Austria; 6 Life and Medical Sciences Institute, Chemical Biology and Chemical Genetics, University of Bonn, Bonn 53115, Germany; 7 Centre of Aptamer Research and Development, University of Bonn, Bonn 53115, Germany

**Keywords:** cell-SELEX, DNA aptamers, chronic kidney disease, inflammatory kidney disease, cytokines

## Abstract

Chronic kidney disease (CKD) is a progressive pathological condition marked by a gradual loss of kidney function. Treatment of CKD is most effective when diagnosed at an early stage and patients are still asymptomatic. However, current diagnostic biomarkers (e.g., serum creatinine and urine albumin) are insufficient for prediction of the pathogenesis of the disease. To address this need, we applied a cell-SELEX (systematic evolution of ligands by exponential enrichment) approach and identified a series of DNA aptamers, which exhibit high affinity and selectivity for cytokine-stimulated cells, resembling some aspects of a CKD phenotype. The cell-SELEX approach was driven toward the enrichment of aptamers that internalize via the endosomal pathway by isolating the endosomal fractions in each selection cycle. Indeed, we demonstrated co-localization of selected aptamers with lysosomal-associated membrane protein 1 (LAMP-1), a late endosomal and lysosomal marker protein, by fluorescence in situ hybridization. These findings are consistent with binding and subsequent internalization of the aptamers into cytokine-stimulated cells. Thus, our study sets the stage for applying selected DNA aptamers as theragnostic reagents for the development of targeted therapies to combat CKD.

## Introduction

The diagnosis of chronic kidney disease (CKD) is currently based on the estimated glomerular filtration rate (eGFR) and urinary albumin excretion. A progressive decrease in the former results in stages G1 (preserved eGFR) to G5 (severely reduced eGFR, often necessitating initiation of renal replacement therapy), and the latter is defined as stages A1 (normal urinary albumin excretion) to A3 (albuminuria in excess of 300 mg/day).^1^ A recent report on the global prevalence of CKD based on stages of glomerular filtration rate was published by Mills et al.^2^ in 2015. The authors estimated that 225.7 million men and 271.8 million women are affected, which clearly indicates that CKD is a major public health problem.

CKD has an important impact at both the patient level, by decreasing quality of life and life expectancy,^3^, ^4^ and at the population level, by increasing health care costs and the demand for health care services.^5^ eGFR and albuminuria reflect glomerular function abnormalities but do not allow investigators to draw conclusions on disease pathophysiology, which is definitely multifactorial. Glomerular pathology is only one component to consider, as many lines of evidence suggest that tubulointerstitial disease is at least as important^6^ and even represents a final common pathway. The histopathology is characterized by an infiltration of inflammatory cells, tubular cell loss, (myo)fibroblast accumulation, and rarefaction of the peritubular vasculature, accompanied by deposition of the interstitial matrix,^7^ and most studies suggest that progression to renal failure correlates more closely with tubular than with glomerular damage.^7^, ^8^, ^9^ As a proof of concept, Grgic et al.^10^ recently developed a unique mouse model of kidney injury and presented evidence that acute injury to the proximal renal tubules is sufficient to produce the full spectrum of pathological changes associated with progressive CKD.

Thus, the proximal tubular epithelial cells are important contributors to the pathophysiology of CKD and therefore represent an especially interesting target for treatment.^11^ It was shown that these cells not only are passive downstream targets of injury induced by molecules, which can pass the injured glomerular filter, but, importantly, per se, contribute to the progression of injury via induction of a pro-inflammatory state. Accordingly, these findings suggest that interventions aimed at proximal signaling events originating from tubules may prevent pathological changes that lead to CKD^10^ and, ideally, therapy would exclusively target tubular cells with an altered phenotype.

To simulate these conditions, we employed a renal proximal tubular epithelial cell (RPTEC) system. RPTEC/TERT1 cells represent a human RPTEC line, immortalized by stable overexpression of the catalytic subunit of human telomerase (TERT1).^12^ These cells resemble the proximal tubular cell type found in vivo, with respect to characteristic morphological and functional properties such as the expression of amino peptidase N, parathyroid hormone (PTH)-sensitive cyclic AMP (cAMP) induction, sodium-dependent phosphate uptake, and other transport activities including glucose and albumin uptake.^12^, ^13^ In addition, RPTEC/TERT1 cells are reported to be stimulated by distinct pro-inflammatory and pro-fibrotic cytokines such as interleukin (IL)-1β, oncostatin M (OSM), or transforming growth factor (TGF)-β1.^14^, ^15^ These factors contribute to the progression of tubulointerstitial fibrosis by mediating a local inflammatory response and/or by affecting a proximal tubular cell phenotype. IL-1β and OSM, for example, represent early and strong stimulators of pro-inflammatory CCL2 mRNA expression in RPTEC/TERT1 cells.^14^

In recent years, aptamers have been sought as a promising tool for various biomedical applications (e.g., in diagnosis, therapy, and biomarker discovery).^16^, ^17^ Aptamers are short single-chained nucleic acids that fold into defined 3D structures that can bind to various ligands. They are identified by an iterative enrichment process, termed SELEX (systematic evolution of ligands by exponential enrichment), and interact with cognate target molecules with high affinity and specificity.^18^ Aptamers bind to a plethora of different target structures (e.g., proteins, small molecules, peptides, or entire cells). The latter seeks to fulfill demands for the generation of molecular targeting tools targeting specific cells and cell types, a need which has emerged in the last decade.

To address this demand, sophisticated cell-SELEX procedures have been developed to enable the enrichment of cell-type-specific aptamers. For example, aptamers specifically recognizing tumor cells, such as lymphoma or prostate cancer, were generated and validated. These aptamers are demonstrated to represent valuable molecular tools for the development of diagnostic and targeted therapy approaches.^17^

Hence, in this study, we employed a cell-SELEX approach and aimed to identify cell-internalizing DNA aptamers that specifically target cells with a pathophysiologically altered phenotype. Following a conventional SELEX procedure, aptamers were isolated specifically from the endosomal fractions of cytokine-stimulated cells, resembling aspects of CKD. By employing this approach, we selected and identified seven DNA aptamers, designated as Apta-1 to Apta-7, which exhibited high affinity and selectivity for the target cells compared to controls. Based on fluorescence in situ hybridization, aptamer candidates exhibit high binding affinity and subsequent internalization into cytokine-stimulated RPTEC/TERT1 cells. Overall, our study demonstrates a novel approach for selection of cell-internalizing DNA aptamers, which are potential molecular probes for diagnosis and/or targeted drug delivery to circumvent proximal tubular injury and thus progression of CKD.

## Results

### In Vitro Selection of DNA Aptamers for CKD

**Figure 1:**
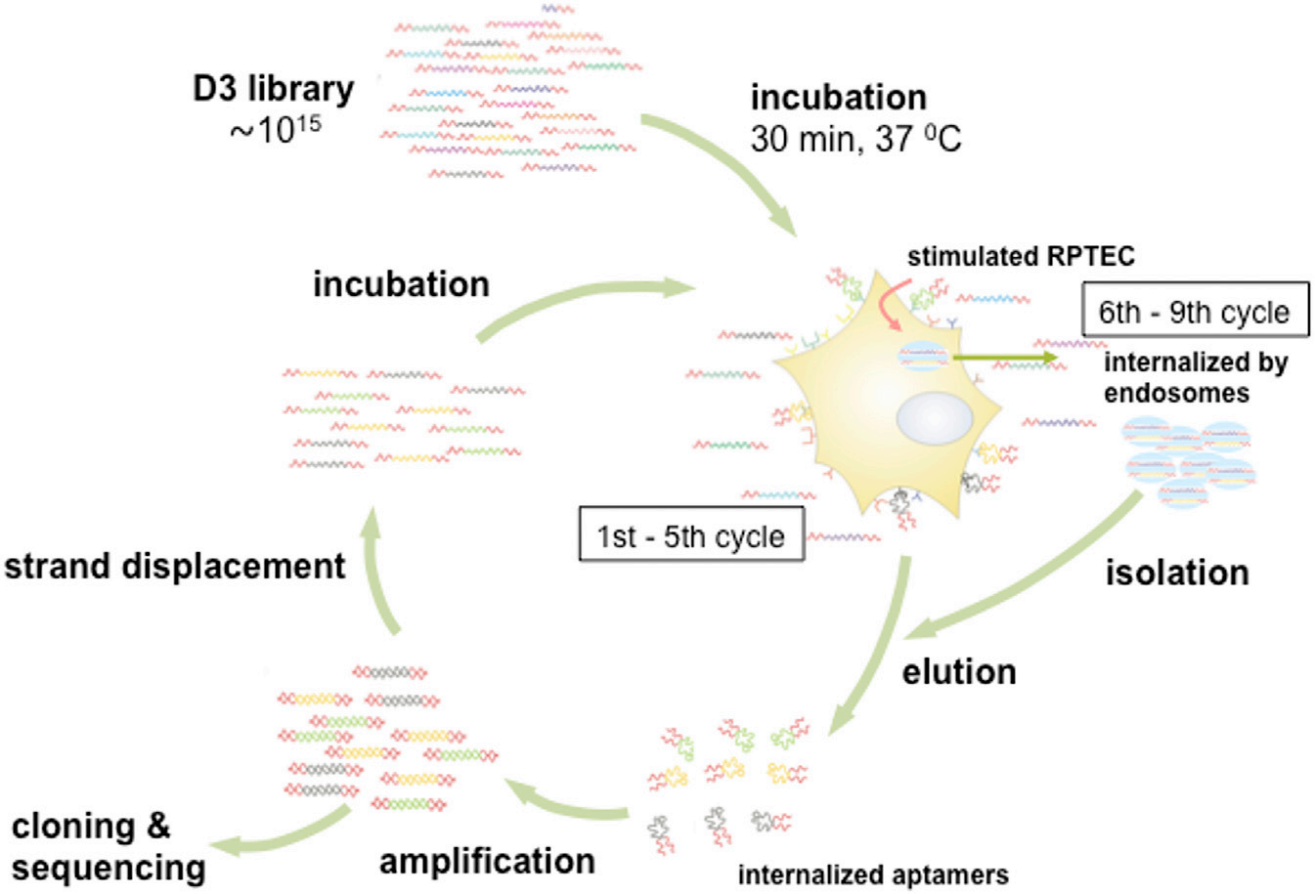
In Vitro Selection Scheme for DNA Aptamers Cytokine-stimulated (CK^+^) renal proximal tubular epithelial RPTEC/TERT1 cells were used for selection. Enrichment of aptamers was assessed by a radioactive binding assay employing CK^+^ cells. Unstimulated (CK^−^) cells were used as an internal control. Following pre-selection, endosome-based selection was performed by cell homogenization followed by continuous sucrose gradient centrifugation. The enrichment process was performed for another four rounds and assessed by a radioactive binding assay. Cell-internalized aptamers were cloned and sequenced from the sixth to ninth rounds of selection.

For in vitro selection, we employed a single-stranded DNA (ssDNA) library consisting of approximately 10^15^ different DNA molecules and containing a 43-nucleotide (nt) randomized region, flanked by 18-nt and 19-nt primer sequences at the 5′ and 3′ ends, respectively (Figure 1). From this library, DNA aptamers that were able to specifically differentiate between cytokine-unstimulated and cytokine-stimulated cells (designated as CK^−^ cells and CK^+^ cells, respectively) were selected.

The renal proximal tubule is one of the primary targets of renal injury, which drives inflammation, tubular atrophy, and the formation of interstitial fibrosis.^8^, ^10^ To this end, for in vitro selection, we employed a RPTEC line (RPTEC/TERT1) as a model system. RPTEC/TERT1 cells were stimulated with a mixture of pro-inflammatory and pro-fibrotic cytokines such as IL-1β, OSM, or TGF-β1, which all have been reported to contribute to the progression of tubulointerstitial fibrosis by mediating a local inflammatory response and/or by affecting the proximal tubular cell phenotype.^14^, ^15^ Subsequently, CK^−^ or CK^+^ cells were employed for selection of DNA aptamers.

In the first six selection cycles, we incubated the DNA library with CK^+^ cells, unbound nucleic acids were removed by washing, and the retained sequences were recovered under denaturing conditions. The recovered sequences were amplified by PCR, and the single-stranded sequences were generated by strand displacement and employed as an input library in the subsequent selection cycle. With increasing selection cycles, more stringent washing conditions and DNase treatment were employed, prior to aptamer recovery, to eliminate aptamers that were only loosely bound to the surface of CK^+^ cells and not taken up by them (Materials and Methods).

**Figure 2:**
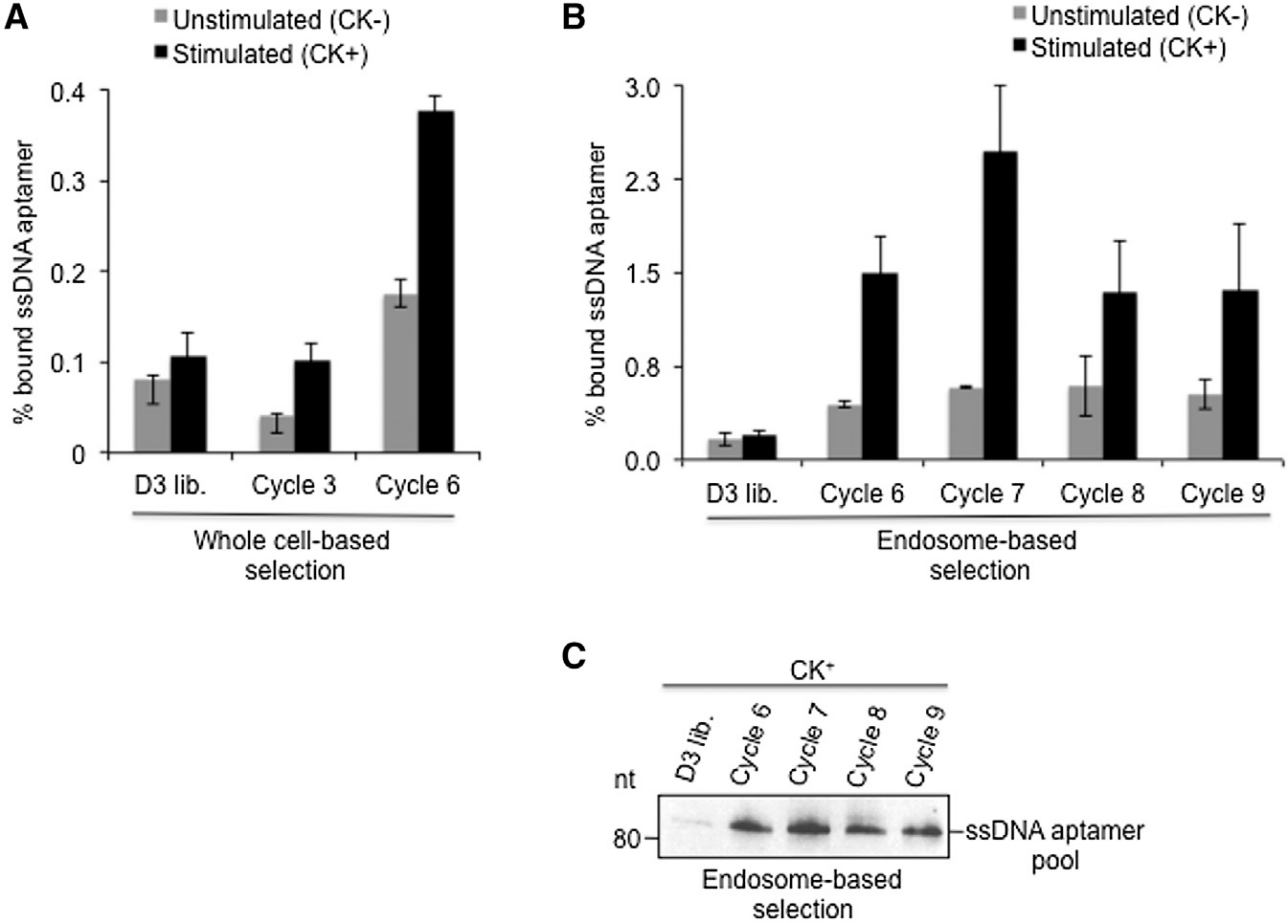
Binding and Internalization of Respective Enriched DNA Aptamer Pools That Target Cytokine-Stimulated Cells (A and B) Binding activities of enriched pools of DNA aptamers, which were either isolated from whole cells (A) or from endosomal vesicles (B) of cytokine-stimulated (CK^+^) cells, were evaluated by a radioactive binding assay. Experiments were performed in three independent trials. Bar graphs represents the mean ± SD. (C) Enriched pools of cell-internalized aptamers from endosomal vesicles of CK^+^ cells (B) were PCR amplified, analyzed on a denaturing polyacrylamide gel, and visualized by ethidium bromide staining.

The enrichment of the DNA library was investigated by radioactive labeling of DNA aptamers obtained from individual selection cycle (see the Materials and Methods). These experiments revealed a selective binding of the enriched DNA libraries to CK^+^ compared to CK^−^ cells (Figure 2A). Specifically, a 2.5-fold and 2.2-fold increase in binding of the DNA library was observed in selection cycles 3 and 6, respectively, compared to 1.3-fold for the starting DNA library. In these analyses, we also observed that levels of tightly bound (and/or subsequently potentially internalized) DNA aptamers to CK^+^ cells increased gradually up to 3.6-fold in selection cycle 6 (0.38%), relative to the starting library (0.11%). Increased binding of the enriched DNA library to CK^−^ cells (0.08% to 0.18%) was also observed, albeit to a lesser extent (2.1-fold) (Figure 2A). This indicates that specific cell surface molecules might be recognized by members of the enriched DNA library, which are also present on unstimulated cells and whose expression level is increased upon stimulation.

### Selection of DNA Aptamers Associated with Endosomal Uptake

The renal proximal tubule of the kidney is a major site where reabsorption of proteins and other substances (e.g., carrier-bound vitamins and trace elements) from glomerular filtrates are facilitated via endocytosis.^19^, ^20^, ^21^ To develop potential molecular probes with high efficiency for not only diagnosis but also potential targeted drug delivery, we thus aimed to preferentially select cell-internalizing DNA aptamers with high specificity to CK^+^ cells. Hence, a more stringent selection process was employed for the successive rounds of selection. To that end, we isolated DNA aptamers in selection cycles 6–9, which were associated with endosomal vesicles within CK^+^ cells, by cell homogenization followed by sucrose gradient centrifugation.^22^

Previous studies reported that most eukaryal cells are able to internalize molecules from their surroundings via different mechanisms (e.g., endocytosis or pinocytosis).^23^, ^24^ Internalization of molecules can be facilitated by endosomal vesicles, which are derived from the invagination of the plasma membrane to form a new intracellular membrane-limited vesicle about 0.05–0.1 μm in diameter.^25^ In receptor-mediated endocytosis, extracellular molecules (the ligands) are internalized selectively through binding with their specific receptor on the plasma membrane. Ligand-receptor complexes incorporated into the intracellular transport vesicles are fused with early endosomes and either sorted for recycling back to the plasma membrane or delivered to late endosomes and lysosomes for degradation.^25^, ^26^

RPTECs facilitate reabsorption of >80% of proteins and other substances from the glomerular filtrates via receptor-mediated endocytosis.^26^ Hence, using an endosome-based selection approach, we specifically enriched for DNA aptamers being internalized via the endosomal pathway, which is known to also transport receptor proteins (e.g., receptor tyrosine kinases, which undergo constitutive endocytosis or internalization into the cell).^27^

In our analysis, the cell-internalized DNA library, isolated from endosomes after each round of selection, was amplified by PCR and employed for the following rounds of selection. Binding analysis of the respective DNA aptamer pool, internalized by CK^+^ cells (Figure 2B) from each selection cycle, showed that the amount of bound DNA aptamers significantly increased in the sixth selection cycle (1.5%) compared to the amount of the unselected DNA library (0.20%; Figure 2B) in CK^+^ cells.

In the seventh selection cycle, the internalized DNA aptamer pool was further enriched (2.5%; Figure 2B), while DNA aptamer binding was reduced to 1.4% for the eighth and ninth selection cycles (1.4%) in respect to the unselected DNA library. This loss of binding might be attributed to the variation in the population of DNA molecules within a library from each selection cycle. The selectivity of the enriched DNA libraries to CK^+^ cells was determined by employing CK^−^ cells in the binding experiment (Figure 2B).

We determined that the amount of bound DNA from selection cycles 6–9 was 2-fold to 5-fold higher in CK^+^ cells (1.4%–2.5%) compared to CK^−^ cells (0.4%–0.6%). The respective endosomal fractions, containing DNA aptamers, were analyzed by western blotting, employing an antibody that recognizes lysosomal-associated membrane protein 2 (LAMP-2), a protein previously reported to be present in late endosomes and lysosomes^28^ (Figure S1). Subsequently, the amplified DNA aptamer pool from each selection cycle was analyzed by denaturing PAGE (Figure 2C).

The DNA libraries from selection cycles 6–9 were cloned and analyzed by Sanger sequencing. Sequences that did not conform to the expected nucleotide length (i.e., 18-base 5′ primer plus 43-base random region plus 19-base 3′ primer) were excluded. In this analysis, we obtained 39, 38, 22, and 28 reads in the sixth, seventh, eighth, and ninth cycles, respectively. Sequence analysis revealed that 67% of the obtained reads harbored G-rich sequences. The reads from the sixth to ninth cycles were pooled, and single copy sequences were eliminated. Subsequently, we selected DNA aptamer candidates exhibiting G-rich or G-quartet core motifs, based on their multiple occurrence from each selection cycle and/or high number of reads.

### Identification and Characterization of ssDNA Aptamers as Molecular Probes for Detection of Surface Markers in Cultured Cells Resembling Aspects of CKD

**Figure 3:**
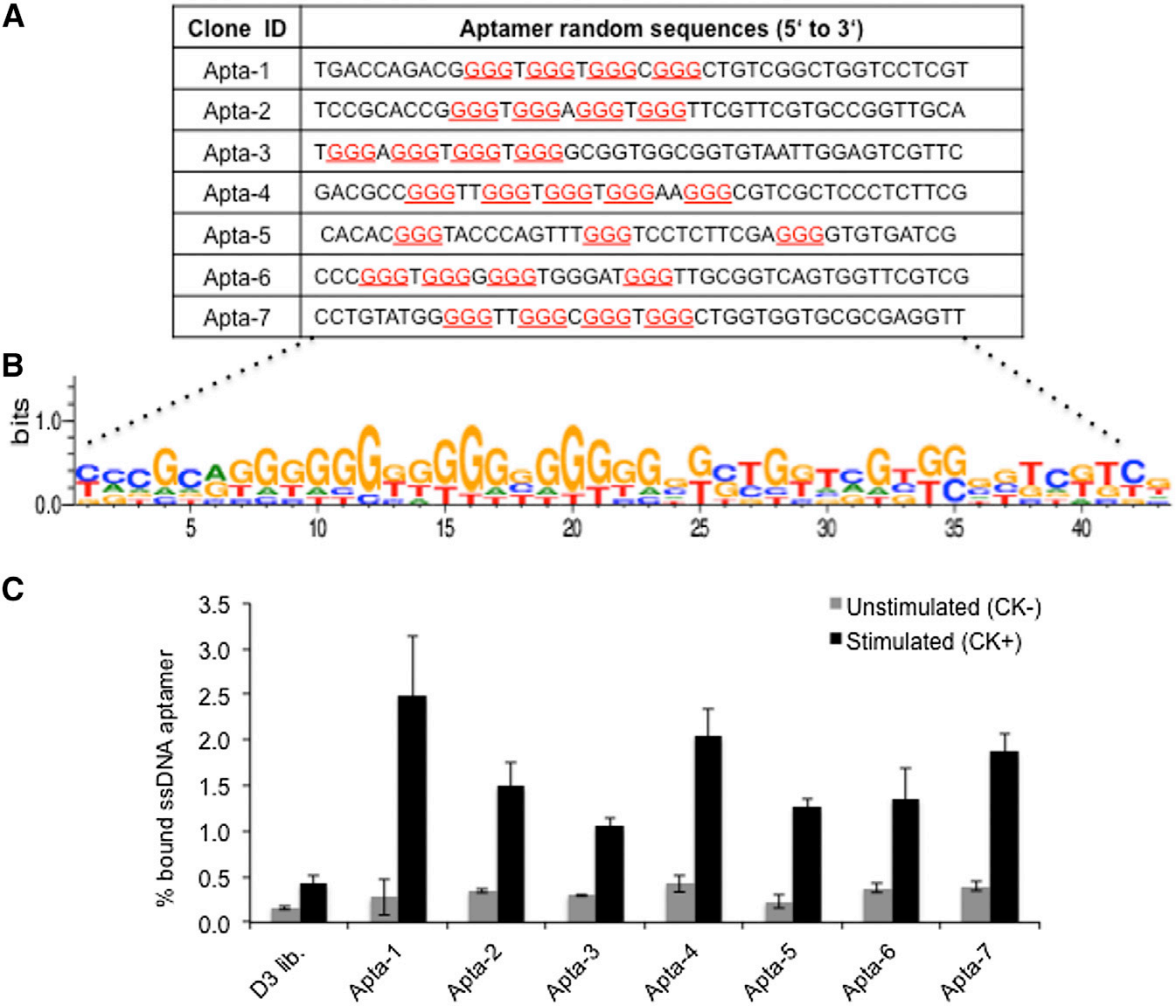
Binding Activities of Selected DNA Aptamers to Cytokine-Stimulated Cells (A) Sequences of individual ssDNA aptamer candidates. G-quartet or G-rich motifs (underlined) within the random sequences (n = 43) of the individual aptamer are indicated. (B) Analysis of the consensus sequences of selected aptamers (A) employing WebLogo software. Results represent stacks of nucleic acid symbols based on a multiple alignment. The height of the symbols within the stack denotes the relative frequency of the nucleic acid, while the overall height of the stack represents the sequence conservation at that position. (C) Relative binding of full-length aptamers (A) labeled with [γ-^32^P]-ATP was analyzed by radioactive binding assay, employing cytokine-unstimulated (CK^−^) or stimulated (CK^+^) cells. Results are presented as the mean ± SD from three independent experiments.

Following the sequence analysis of the DNA libraries from the sixth to ninth selection cycles, which were obtained from endosomal fractions of CK^+^ cells, we identified seven aptamer candidates (Figure 3A) that showed selective binding and/or internalization into CK^+^ cells (Figure 3C). By examining the randomized region of the respective aptamers, we observed an increased occurrence of guanine residues within the selected region of the aptamers (i.e., Apta-1, Apta-2, Apta-3, Apta-4, Apta-5, Apta-6, and Apta-7, respectively), potentially forming G-quadruplex structures (except for Apta-5, which lacked this sequence motif). The guanine-rich (G-rich) consensus sequence of all identified aptamers (Figure 3B) was verified by WebLogo software.^29^

Previous studies reported that G-rich DNA sequences can assemble into four-stranded tertiary structures, designated as G-quadruplexes^30^ with a predicted sequence motif of d(G3+N1-7G3+N1-7G3+N1-7G3+).^31^ G-quadruplex structures are formed by layers of square planar of guanines (G-quartets or tetrads) stabilized by Hoogsteen hydrogen bonding, while their formation is induced by monovalent cations at physiological conditions.^32^ Rhodes and Lipps^32^ reported that G-quadruplexes are able to form parallel or anti-parallel structures, depending on the orientation of the G-quartet in a strand, and exert essential roles in different biological processes (e.g., DNA replication, gene expression, and telomerase maintenance).

To determine the selectivity and relative binding activity of DNA aptamers to CK^+^ cells, we performed a radioactive binding assay employing chemically synthesized DNA aptamers. Selective binding and internalization of aptamers was assessed by employing CK^−^ or CK^+^ cells, respectively. All individual aptamers exhibited selective binding to CK^+^ versus CK^−^ cells, showing a 4- to 9-fold increase in bound aptamers to CK^+^ cells (0.97% to 2.5%) compared to CK^−^ cells (0.22% to 0.42%; Figure 3C). The fold change of bound aptamer in CK^+^ cells with respect to CK^−^ cells was highest for Apta-1 (9-fold), followed by Apta-5 (6-fold), Apta-4 and Apta-7 (5-fold), and Apta-2, Apta-3, and Apta-6 (4-fold), respectively. Importantly, a significantly higher binding affinity was observed for all individual aptamers relative to the corresponding DNA library from which they were derived. These data suggest that all individual aptamers exhibit increased binding to and/or internalization into CK^+^ cells and thus might recognize biomarkers present in injured renal proximal tubular cells.

### Predicted Secondary Structures of Selected Cell-Internalizing DNA Aptamers Containing a G-Quartet Motif

G-quadruplexes represent highly stable structures with a common folding motif and they are polymorphic, since they can exhibit different strand configurations (e.g., a parallel or anti-parallel strand configuration with different sequence combinations from t-loop and tail strands). Despite the diversity of G-quadruplex structures, they can elicit specific target recognition of many molecules with a similar scaffold.^33^ It is noteworthy that the G-quartet motif of all individual DNA aptamer candidates, except Apta-5, (Figure 3A) exhibits tail strands and loops of different lengths and sequence combinations, yet all of these selected aptamers confer target recognition to CK^+^ cells, some of which even display very similar binding activities. In addition, Apta-5, which contains G-rich sequences but lacks the G-quartet sequence motif, also exhibited significant binding activity. These observations may not only reflect the conformational plasticity of selected aptamers containing the G-quartet motif, but they might also be due to the presence of various target sites on CK^+^ cells, recognized by the different aptamers.

We analyzed the predicted secondary structures of all individual aptamer candidates to identify potential secondary structure binding motifs that elicit target binding on CK^+^ cells, employing m-fold software.^34^ The primer sequences were included in sequence-structure motif prediction, as they might play a role in folding and target binding of aptamers. Analysis of the secondary structure of aptamer candidates revealed that each aptamer forms a unique structure (Figure S2). Within the 2D structures, the G-quartet motif of Apta-1, Apta-3, Apta-4, and Apta-6 is located within a loop, whereas the G-quartet motif of Apta-2 and Apta-7 is part of a loop and stem structure. However, whether the 2D structures, predicted by m-fold, might indeed be present has to be elucidated in future experiments; in particular, since, due to the presence of G-quartet motifs, alternative 2D structures, not predicted computationally by the m-fold program, might be formed.

### Minimal Sequence Motif of Apta-1 Binding to Cytokine-Stimulated Cells

Since all individual aptamers, except one, harbored G-rich stretches and/or represented potentially G-quadruplex-forming structures, we wanted to elucidate the minimal length of the G-quartet motif, which allows efficient binding/internalization to CK^+^ cells. To that end, we deleted the constant regions from the Apta-1 aptamer, resulting in a 43-nt randomized region, and/or shortened the selected random region to 32 nt, containing the G-quartet motif only (Figure S3A). Removal of the constant regions, however, largely abolished binding of Apta-1 to CK^+^ cells (Figure S3B), indicating that these regions might add significantly to target binding of Apta-1 to CK^+^ cells.

Although the roles of G-quadruplex loops and tail strands have not yet been clearly elucidated, until now, previous studies have indeed shown that the loops and tail strands are able to influence the G-quadruplex interactions with target molecules, including their stability and structural conformation and thus their target specificity.^35^, ^36^ Because the head and tail sequences of G-quartet motif are obviously required, only full-length aptamers were employed for all of the following experiments.

### Surface Binding of DNA Aptamers to Cytokine-Stimulated Cells

The target recognition of ssDNA aptamers (i.e., cellular membrane receptors) is attributed to their ability to form unique 3D structures.^37^, ^38^ Through specific interaction, aptamers might subsequently be internalized via receptor-mediated endocytosis and trapped by endocytic vesicles, and they can also undergo endosomal escape or release.^38^ In the proximal tubule, reabsorption of proteins and other molecules from the glomerular filtrates is mainly facilitated by receptor-mediated endocytosis, which is mediated by megalin and cubilin membrane receptors under normal conditions. Indeed, efficient reabsorption of proteins and substances from glomerular filtrates is a vital function of RPTECs.^19^, ^21^

Various cell surface receptors are upregulated in response to renal injury, such as TGF-β1,^39^ kidney injury molecule-1 (KIM-1),^40^, ^41^ angiotensin type I (AT1R),^42^ and urokinase,^43^ leading to interstitial inflammation and renal fibrosis. The megalin receptor represents also a key factor in tubulointerstitial injury, as it facilitates increased endocytosis of glomerular filtrate products in RPTECs, leading to activation of pro-inflammatory cytokines.^44^

**Figure 4:**
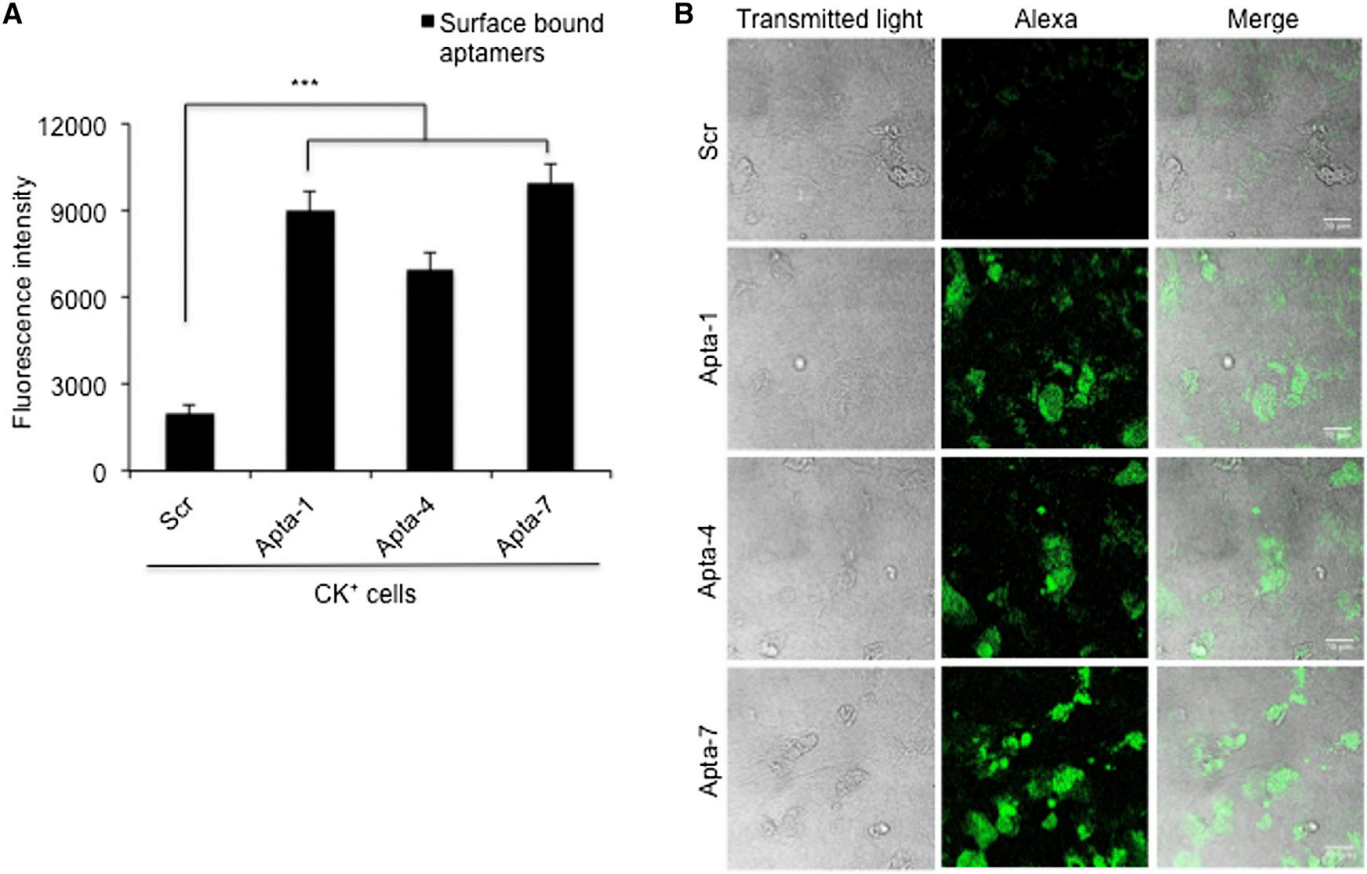
Surface Binding of Selected Aptamers—Apta-1, Apta-4, and Apta-7—to Cytokine-Stimulated Cells (A) Cytokine-stimulated (CK^+^) cells were fixed with paraformaldehyde and incubated with 50 nM AlexaF488-labeled full-length DNA aptamers (Apta-1, Apta-4, Apta-7, or scrambled [Scr] sequence, respectively) for 30 min at 37°C. Surface-bound aptamers were quantified by a fluorescence-based detection assay employing a plate reader. The experiment was performed in triplicate from two independent trials. The bar graph represents the mean ± SEM. Statistical analysis was performed by using the Student t test. ***p < 0.001. (B) CK^+^ cells, which were incubated with aptamers as in (A) under comparable conditions, were analyzed by confocal imaging. Green fluorescein signal indicates surface-bound aptamers. Scale bars represent 20 μm.

Therefore, to examine whether the observed binding activity of the aptamer candidates is mediated by surface biomarkers, which might be upregulated in CK^+^ cells, we performed a surface binding assay. Hence, selected DNA aptamer candidates (Apta-1, Apta-4, and Apta-7, respectively) exhibiting the highest binding activity were used for surface staining of CK^+^ cells. To that end, cells were fixed with paraformaldehyde and subsequently treated with the Alexa Fluor-labeled aptamers. The amount of surface-bound aptamer was determined by a fluorescence intensity-based assay employing a plate reader. Indeed, increased surface binding was determined for Apta-1 (4.5-fold), Apta-4 (3.5-fold), and Apta-7 (5-fold), compared to a scrambled control sequence (Scr) of Apta-1 (Figure 4A).

In parallel, we also performed confocal imaging under similar binding conditions. As expected, confocal imaging analysis of CK^+^ cells showed that the fluorescence intensity signal of Apta-1, Apta-4, and Apta-7 was more pronounced than that of the scrambled sequence (Figure 4B). These results are consistent with the increased expression of cell surface markers in CK^+^ cells (see above) in response to IL-1β, OSM, and TGF-1β stimulation. This might also explain the observed binding specificity of selected aptamer candidates to CK^+^ cells. In addition, these biomarkers (which might serve as target recognition sites of the aptamer candidates) may mirror the biomarkers present in injured RPTECs in patients with CKD.

### Internalization of DNA Aptamers via the Endosomal Pathway

**Figure 5:**
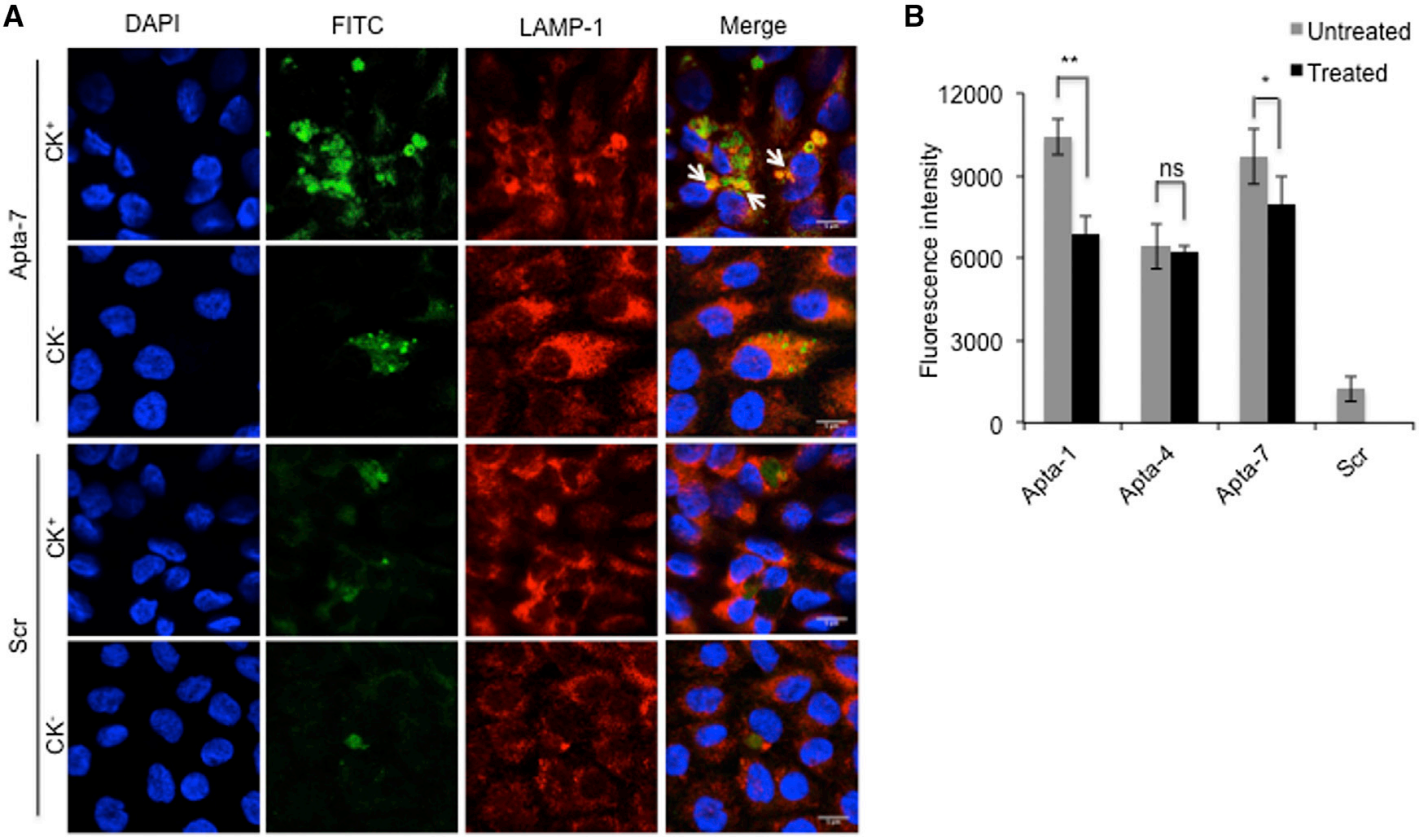
Internalization of G-Rich Aptamer Candidates Is Facilitated by Endosomal Vesicles (A) Cytokine-unstimulated (CK^−^) or stimulated (CK^+^) cells were incubated with 100 nM of either FITC-labeled (green) Apta-7 or a scrambled sequence (Scr) as an internal control. Co-localization of Apta-7 with the late endosomal and lysosomal marker LAMP-1 (red) was analyzed by confocal imaging. Nuclear staining (blue) was performed employing DAPI. (B) CK^+^ cells were either left untreated or treated with 200 μM chloroquine, a reported inhibitor of clathrin-mediated endocytosis, for 2 hr. Following chloroquine treatment, cells were incubated with AlexaF488-labeled full-length aptamers (Apta-1, Apta-4, Apta-7, or a scrambled sequence). Cells were washed with acid and fixed with paraformaldehyde, and fluorescence signals were determined employing a plate reader. The experiment was performed in triplicate. The bar graph represents the mean ± SD. Statistical analysis was performed by using the Student t test. *p < 0.05, **p < 0.01. Scale bard represent 5 μm. ns, non-significant.

Since the aptamer candidates were selected specifically from endosomal fractions of CK^+^ cells, we aimed to demonstrate that internalization of aptamers is indeed mediated by endosomal vesicles. To that end, we analyzed the co-localization of fluorescein isothiocyanate (FITC)-labeled Apta-7 and LAMP-1, a marker for late endosomes and lysosomes, in CK^+^ or CK^−^ cells, respectively (Figure 5A). A scrambled DNA sequence was used as a negative control. Compared to the scrambled sequence, cellular internalization of Apta-7 was observed, which co-localized with LAMP-1 (predominantly in the perinuclear region of CK^+^ cells), thereby indicating similar distribution of Apta-7 and late endosomes and/or lysosomes.

To further examine the cellular uptake of Apta-7, we performed a time-course binding experiment employing ATTO-labeled Apta-7 or a scrambled sequence as a control. An increased amount of internalized Apta-7 was detected with an increasing incubation period, whereas almost unchanged levels of the scrambled DNA control were detected throughout the incubation period (Figure S4A). Furthermore, the cellular uptake of Apta-7 was higher than that of the Scr for all of the time points indicated (i.e., 5.6-fold at 15 min, 5.0-fold at 30 min and 7.9-fold at 60 min, respectively), suggesting that cellular uptake of Apta-7 is regulated by an endosomal pathway.

In RPTECs, filtered proteins are mainly reabsorbed via clathrin-mediated endocytosis involving megalin and cubilin receptors,^45^ and the regulation of this pathway involves two NPXYs motifs that sequester protein complexes (i.e., clathrin and AP-2), which are involved in coated pit formation.^19^, ^46^ Clathrin-coated vesicles (CCVs) facilitate internalization of ligand-receptor complexes by fusing with early endosomes. This pathway involves endosomal luminal acidification that facilitates ligand-receptor dissociation, ligand processing, receptor recycling or degradation, vesicular trafficking, and fusion to late endosomes and lysosomes.^26^

To determine whether CCVs facilitate internalization of the aptamer candidates, we performed an inhibition assay employing chloroquine, a known inhibitor of clathrin-mediated endocytosis.^47^ Cytokine-stimulated or CK^+^ cells were either untreated or treated with chloroquine inhibitor for 2 hr prior to incubation of AlexaF488-labeled aptamers (Apta-1, Apta-4, and Apta-7). Following aptamer binding and internalization, cells were washed with acid to remove surface-bound aptamers, fixed with paraformaldehyde, and analyzed employing a plate reader. Analysis of fluorescence signals showed that chloroquine treatment significantly reduced the internalization of Apta-1 (p < 0.001) and Apta-7 (p < 0.01), but not Apta-4 (p > 0.05) (Figure 5B), relative to the untreated cells.

Although these data suggest that clathrin may indeed play a role in the internalization of Apta-1 and Apta-7, respectively, they also indicate that other vesicles and/or mechanisms are involved in regulating endocytosis of the remaining aptamer candidates, as in the case of Apta-4, thereby indicating that the selected aptamers might interact differently with surface markers.

### Specificity and Binding Affinity of DNA Aptamer Candidates to Cytokine-Stimulated Cells

Initially, we tested the specificity of selected aptamers such as Apta-7 by employing additional control cells using differentiated cells only (designated as CK^−/−^) for binding/internalization experiments. We examined the binding activity of ATTO-labeled Apta-7 in CK^−/−^, CK^−^, and CK^+^ cells, respectively, and compared it to the binding activity of the scrambled sequence (Figure S4B). As expected, an Apta-7 fluorescence signal was significantly increased in CK^+^ cells compared to a scrambled sequence. A residual fluorescence signal of Apta-7 was also detected in CK^−^ cells but was surprisingly not detectable in CK^−/−^ cells, whereas comparable amounts of an Scr were detected for all cell conditions. These data are consistent with the presence of shared target binding sites in CK^−^ and CK^+^ cells, which are possibly induced in response to cellular stress caused by starvation (as also shown in Figure 5A), and demonstrate the specificity of the selected aptamer to CK^+^ cells.

Binding of Apta-7 was also investigated in other cell lines that were either non-stimulated or stimulated with cytokines. Under non-stimulated conditions, Apta-7 showed higher binding to HeLa (human cervical cancer) cells than to MCF-7 (human breast cancer) cells. A weak binding signal of Apta-7 was also observed in N2A (mouse neuroblastoma) cells and undifferentiated RPTECs (Figure S4C). Binding of Apta-7 to HeLa and MCF-7 cells might be attributed to the presence of similar or identical target molecules recognized by Apta-7.

The specificity of Apta-7 was also examined in these cell lines under stimulated conditions. We show that binding of Apta-7 was enhanced in stimulated cells (Figure S4D) when compared to non-stimulated cells (Figure S4C) for all cell lines. Importantly, our data revealed that the specificity of Apta-7 is clearly higher in CK^+^ (5-fold) than in N2A (2-fold), MCF-7 (3-fold), or HeLa cells (2-fold), respectively, suggesting that Apta-7 predominantly recognizes target molecules in CK^+^ cells compared to other tested cell lines.

### Binding Constants of Selected Aptamers to Cytokine-Stimulated Cells

**Figure 6:**
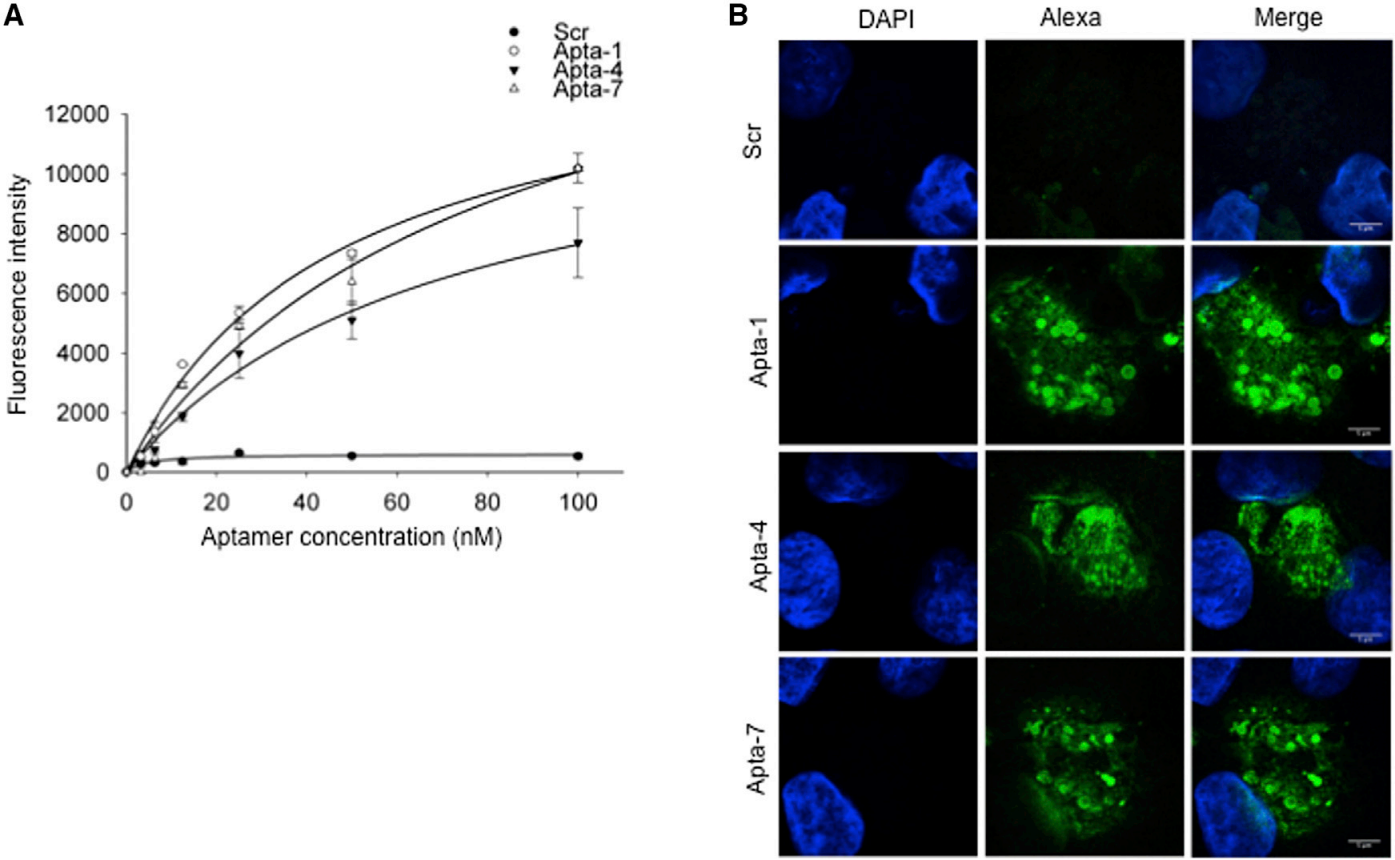
Binding Specificity and Affinity of Cell-Internalizing Aptamer Candidates—Apta-1, Apta-4, and Apta-7—to Cytokine-Stimulated Cells (A) Apparent binding affinity constants (Kd_app_) of AlexaF488-labeled full-length aptamers (Apta-1, Apta-4, and Apta-7) to cytokine-stimulated (CK^+^) cells were determined by fluorescence intensity measurement. A scrambled sequence (Scr) was used as an internal control. Cells were incubated with increasing concentrations of aptamers (i.e., from 0 to 100 nM) for 30 min at 37°C. Subsequently, cells were washed and fixed with paraformaldehyde, and fluorescence signals of bound and internalized aptamers were analyzed employing a plate reader. The fluorescence intensity mean values were fitted employing SigmaPlot software to determine the apparent binding affinity constants of selected aptamers. The experiment was performed in triplicate from two independent trials. The bar graph represents the mean ± SE calculated from the SigmaPlot. (B) Confocal image analysis of CK^+^ cells treated with 50 nM AlexaF488-labeled full-length aptamers. The experiment was performed as in (A) under comparable conditions (i.e., buffer and incubation condition). Green fluorescent signals indicate aptamers bound/internalized into cells. Blue signals indicate nuclear DAPI staining. Scale bars represent 5 μm.

To determine the apparent binding affinity constant (Kd_app_) of Apta-1, Apta-4, and Apta-7 in stimulated RPTEC/TERT1 (CK^+^) cells, we performed a fluorescence-based binding assay. Different titers of AlexaF488-labeled aptamers (i.e., from 0 to 100 nM) were incubated with CK^+^ cells for 30 min in standard cell culture conditions. The Kd_app_ was calculated using SigmaPlot software (https://systatsoftware.com/products/sigmaplot/product-uses/sigmaplot-product-uses-analysis-of-ligand-binding-data/). The specificity and binding curves of selected aptamer candidates are shown in Figure 6A. The binding assay demonstrates that Apta-1 exhibits the highest binding affinity, followed by Apta-4 and Apta-7, based on their calculated apparent affinities of 46 ± 4, 69 ± 27, and 82 ± 19 nM, respectively. A significant increase in fluorescence intensity from 12.5 nM to 100 nM was observed for the three aptamers compared to the fluorescence signals of the scrambled sequence.

Comparable buffer conditions were employed, and binding and internalization of selected aptamer candidates was assessed by confocal imaging (Figure 6B). Confocal image analysis showed that the fluorescence signals, detected in CK^+^ cells incubated with either AlexaF488-labeled Apta-1, Apta-4, or Apta-7, respectively, were significantly increased compared to the cells incubated with an Scr, thus confirming the specificity of aptamer binding to CK^+^ cells. Using confocal microscopy, we also observed that the subcellular localization of all of the aptamer candidates resided mainly in the cytoplasm.

## Discussion

Early diagnosis and therapeutic intervention remains a crucial and a major challenge in patients with CKD. Since biomarkers for early diagnosis of CKD (i.e., proximal tubular injury) are currently largely missing, we selected specific DNA aptamers for an improved diagnosis of CKD by a cell-SELEX approach. To that end, a selection process for isolating cell-internalizing DNA aptamers with high specificity and affinity to CK^+^ cells was established.

We performed the cell-SELEX selection first by a whole-cell-based selection, followed by an endosome-based selection. With this approach, we were able to identify seven aptamers, internalized into cells by an endosomal pathway, exhibiting high specificity for CK^+^ cells. This approach also favors isolation of aptamers that bind to the native folding and/or modification state (e.g., glycosylation, phosphorylation, etc.) of potential target molecules^17^, ^48^, ^49^, ^50^ over other selection approaches. These aptamers might be employed to identify novel cell surface markers (e.g., receptors), which subsequently become internalized into cells via the endosomal pathway, and to elucidate the mechanisms underlying CKD progression.

As the selection process was performed within a short time period (i.e., within 30 min), this suggests that selected aptamers exhibit rapid binding and internalization into cells. We verified that the internalization of selected aptamers was facilitated by endosomal vesicles, employing late endosome/lysosome markers LAMP-2 and LAMP-1 for western blot analysis and confocal imaging, respectively.

Based on our findings, we envisage the use of these aptamers as potential molecular tools for CKD, since they display specific recognition to target sites and penetration into CK^+^ cells. Based on their sequence, selected aptamers harbor G-rich or G-quartet motifs that have high propensity to form G-quadruplexes known to be implicated in different biological processes (e.g., cellular and viral replication or transcriptional regulation).^51^, ^52^, ^53^ Previous studies also showed that G-quadruplex structures have clinical applications (e.g., in the treatment of cancer or HIV infection, as thrombin inhibitors, or in the sensing of viruses).^52^, ^54^, ^55^ This supports the idea that selected aptamer candidates may also have potential clinical applications. As depicted in binding and confocal image analyses, we show that these aptamer candidates elicit high specificity and affinity to target molecules, which are displayed on the cell surface of CK^+^ cells.

Diagnostic applications of selected aptamers might be exerted as follows: subsequent to injection into organisms, nucleic acids are rapidly cleared from the blood via the kidney.^56^, ^57^ Thus, it can be envisioned that selected aptamers might display an increased retention time upon intravenous injection into patients with CKD compared to healthy controls, which can subsequently be analyzed by PCR analysis of the urine (in which the amount of the secreted aptamer in the urine is compared to an Scr, not retained by the kidney). In addition, the identified aptamers might be employed not only for CKD diagnosis but could also be used in therapeutic approaches by targeting drugs to injured renal tubule tissues of patients with CKD through covalent attachment to selected aptamers.

Indeed, RPTECs have been the target of various treatment approaches. In 1996, Oberbauer et al.^58^ used an anti-sense oligonucleotide for in vivo suppression of the renal Na^+^/Pi cotransporter by hybridizing a 20-mer phosphorothioate oligonucleotide to the message for the rat kidney sodium phosphate cotransporter NaPi-2 close to the translation initiation site, exerting anti-sense effects in the renal proximal tubule. The sodium-glucose cotransporter 2 (SGLT2) inhibitor empagliflozin was recently tested in a prospective randomized placebo-controlled study in a large group of patients with type 2 diabetes mellitus. These drugs inhibit the reabsorption of glomerular filtered glucose by proximal tubular epithelial cells. Empagliflozin, when added to standard glucose-lowering therapy, significantly reduced cardiovascular mortality^59^ and progression of renal disease.^60^ Thus, our study may provide a new prototype by applying selected DNA aptamers as diagnostic tools and/or carriers for delivery of drugs for treatment of renal proximal tubule injury associated with CKD.

## Materials and Methods

### Cell Culture Reagents and Antibodies

Cell culture reagents were purchased from Gibco (Life Technologies). Other materials and reagents were obtained as follows: OSM (Sigma), IL-1β (R&D Systems), TGF-β1 (PeproTech), anti-LAMP-1 and anti-LAMP-2 antibodies (BD PharMingen), random DNA library (D3), aptamers and primer sequences (Integrated DNA Technologies), and glass-bottom 24-well plates (Eppendorf). All other reagents were obtained from Sigma unless otherwise indicated.

### Cell Culture

Renal proximal tubule RPTEC/TERT1 cells were cultured as previously described.^12^, ^14^ Briefly, cells were grown in a serum-free mixture of DMEM/F-12 (1:1) medium containing ITS [insulin (5 μg/mL), transferrin (5 μg/mL), selenium (5 ng/mL)], GlutaMAX (2 mM), epithelial growth factor (EGF) (10 ng/mL), hydrocortisone (36 ng/mL), and penicillin (100 U/mL)/streptomycin (100 μL/mL) at 37°C in a humidified 5% CO_2_ atmosphere. Cells were fed every 2–3 days and grown to a confluent state for 10 days. Subsequently, cells were made quiescent by incubation in serum- and supplement-free medium containing penicillin/streptomycin only for 48 hr. Following incubation, cells were either left unstimulated (CK^−^) or were stimulated (CK^+^) with TGF-β1 (10 ng/mL), IL-1β (10 ng/mL), and OSM (10 ng/mL) for another 48 hr. Cells were washed twice with 1× PBS prior to the selection procedure or binding experiments.

### Cell-SELEX Selection Process

The sequence of the DNA library used for selection was 5′-GCTGTGTGACTCCTGCAA-N_(43)_-GCAGCTGTATCTTGTCTCC-3′. The library contained ∼10^15^ random sequences, flanked by two constant priming sequences at the 5′ and 3′ ends, respectively. The whole-cell selection process was carried out for six cycles. The library input used for the initial cycle was 1 nmol. In the following cycles, the library input was subsequently decreased to 640 pmol for the second cycle, was then decreased to 166 pmol for the third and fourth cycles, and was reduced to 83 pmol for the fifth and sixth cycles, respectively. The selection process was previously described in detail.^16^ Briefly, prior to selection, the library was diluted in SELEX binding buffer (SBB) containing 0.5 mg/mL BSA, 0.5 mg/mL salmon sperm DNA, 1 mM CaCl_2_, and 1 mM MgCl_2_ in 1× PBS and was subsequently boiled at 95°C for 5 min. The library was cooled on ice for 10 min. Prior to incubation, CK^+^ cells were washed twice with 1× PBS and then added to the library for 30 min at standard cell culture conditions. Following incubation, cells were washed with SELEX washing buffer (SBB solution without salmon sperm DNA) and subsequently treated with DNase. The stringency of selection was increased by increasing the amount of DNase (50–200 U), and the number and volume of washing steps (2–4 times) in the succeeding selection cycles.

For DNA aptamer extraction, the phenol-chloroform-isoamyl alcohol (PCI) extraction method was employed. Cells were trypsinized, and the cell suspension was boiled for 5 min at 95°C. One volume of PCI was added to the cell suspension, and the mixture was vortexed vigorously and centrifuged for 5 min at 13,000 × *g*. The upper phase was transferred into another tube; subsequently, one volume of chloroform was added. The mixture was vigorously vortexed and centrifuged for 5 min at 13,000 × *g*. The upper phase was passed through a Sephadex G25 column, and the filtrate was added with a one-tenth volume of 3 M sodium acetate and two volumes of 100% ethanol. Subsequently, the reaction mixture was incubated at −80°C for 20 min. Aptamer precipitates were centrifuged for 30 min at 13,000 × *g*. The pellet was washed with 70% ethanol, air dried, and re-suspended in 100 μL DNase-free water.

For the amplification, eluted DNA aptamers were amplified by PCR using the following oligonucleotides: 5′-FAM-GCTGTGTGACTCCTGCAA-3′ (sense oligonucleotide) and 5′-GGGCGATCGTAAGATCGCCC-spacer C18 (HEG)-GGAGACAAGATACAGCTGC-3′ (anti-sense oligonucleotide). Thermal cycling conditions were as follows: initial 5-min denaturation at 95°C, 16–24 cycles of denaturation (95°C, 1 min), annealing (64°C, 1 min), elongation (72°C, 1.5 min), and final elongation (72°C, 7 min). PCR products were verified by agarose gel electrophoresis; subsequently, the sense DNA strands were isolated by strand displacement, employing polyacrylamide gel electrophoresis on an 8% denaturing gel. The 5′ FAM (6-carboxyfluorescein)-labeled sense DNA strands can be visualized by UV light and separated from the anti-sense strands containing a C18 spacer (HEG), which adds a hairpin structure and migrates more slowly than the sense strands. Subsequently, a pool of sense DNA strands was eluted from the gel and re-suspended in DNase-free water. Selected pools of aptamers were prepared for the next round of selection.

To monitor the enrichment of aptamers, a radioactive binding assay was performed. An isolated pool of sense DNA strands was amplified employing an unmodified sense oligonucleotide: 5′-GCTGTGTGACTCCTGCAA-3′ and a spacer C18 (HEG)-containing anti-sense oligonucleotide 5′-GGGCGATCGTAAGATCG CCC-spacer C18 (HEG)-GGAGACAAGATACAGCTGC-3′. PCR products were purified and analyzed by PAGE to isolate the unmodified sense DNA strands, which were labeled with gamma-ATP-^32^P [γ-^32^P] (Hartmann Analytics) using T4 polynucleotide kinase (NEB). Radioactively labeled aptamers were used for binding assay employing CK^+^ and CK^−^ cells (see details below). The enrichment of the selected aptamer pool was monitored every three cycles.

Following whole-cell-based selection, we aimed to enrich cell-internalized DNA aptamers. Thus, we performed endosome-based selection following the fifth selection cycle. Here, selection was performed on a larger scale using a 10-cm cell culture plate; thus, the amount of the aptamer pool was upscaled by a factor of six. Endosome-based selection (see details below) was performed for another four cycles, and the amount of aptamer used was 500 pmol for the sixth and seventh cycles, which was reduced to half (250 pmol) for the eighth and ninth cycles. The enrichment of cell-internalized aptamers was also examined by radioactive binding assay (see details below).

For identification of individual cell-internalized DNA aptamers, pools of aptamers from the sixth to ninth cycles were prepared for cloning and sequence analysis. Initially, an eluted pool of sense DNA strands from each cycle was PCR amplified using unmodified primers as follows: sense oligonucleotide 5′-GCTGTGTGACTCCTGCAA-3′ and anti-sense oligonucleotide 5′-GGAGACAAGATACAGCTGC-3′. PCR products were purified using the QIAquick PCR Purification Kit (QIAGEN). Purified PCR products were then used for cloning employing the CloneJET PCR Cloning Kit (Fermentas). Generated constructs were used for transformation of *Escherichia coli* competent cells (Invitrogen), which were subsequently plated on ampicillin-resistant yeast extract tryptone (YT)-agar plates. Isolated clones were subjected for colony PCR in a 98-well plate format. PCR products were randomly selected and verified on a 2% agarose gel. Purified PCR products were analyzed by Sanger sequencing (ABI Prism 3100-Sequencer; Life Technologies) and sequences were analyzed by SeqMan software.^61^

### Isolation of Endosomes

Isolation of endosomes was performed as described by de Araùjo et al.,^22^ with a few modifications. Briefly, CK^+^ cells, incubated with aptamers, were washed three times with cold 1× PBS; , 2.5 mL 1× PBS containing a protease inhibitor was subsequently added. Cells were harvested by careful scraping and were subsequently transferred to a 15-mL Falcon tube and centrifuged at 112 × *g* for 5 min at 4°C. Cell pellets were washed in homogenization buffer (HB) (250 mM sucrose and 3 mM imidazole, pH 7.4) containing protease inhibitors (HB+) and were then centrifuged at 700 × *g* for 10 min at 4°C. Cells were re-suspended gently in 200 μL HB+ buffer and homogenized by pipetting the cell suspension back and forth through a 22-gauge needle. Homogenization efficiency, indicated by intact nuclei, was verified by microscopy. Homogenized cells were subsequently centrifuged at 1,000 × *g* for 10 min at 4°C to separate the nuclei pellet from the post-nuclear supernatant (PNS). The sucrose concentration in the PNS was adjusted to 40%–41% using 62% sucrose solution. The PNS was loaded into a SW41 centrifuge tube and overlaid with 7 mL 35% sucrose solution. HB+ buffer was then added to fill the tube. The sample was centrifuged at 197,000 × *g* for 3 hr at 4°C. Following centrifugation, the endosomal fraction (indicated by a milky band formed at the interphase) was collected for DNA extraction as described previously. Prior to DNA extraction, an aliquot of the endosomal fraction was taken for western blot analysis to verify the presence of endosomal vesicles, employing anti-LAMP-2 antibody.

### In Vitro Binding Assays

Aptamer binding and uptake was investigated by employing either radioactive- or fluorescein-labeled aptamers. For radioactive binding assay,^16^ 10 pmol of a pool or of an individual aptamer was labeled at the 5′ end with [γ-^32^P]-ATP (Hartmann Analytics) using T4 polynucleotide kinase (NEB), according to the manufacturer’s instructions. 10 μL dH_2_O was added to the reaction mixture and subsequently purified on a Sephadex G25 column. The eluate was added to a tube containing 1 mL SBB solution, boiled for 5 min at 95°C, and cooled for 10 min on ice. Prior to incubation with aptamers, CK^+^ and CK^−^ cells were washed twice with 2 mL pre-warmed 1× PBS. Cells were subsequently incubated with radioactively labeled aptamers for 30 min at standard cell culture conditions. Following incubation, the supernatant containing the unbound aptamers was transferred into a scintillation bottle. Cells were washed twice with 2 mL SELEX washing buffer (SBB without salmon sperm DNA), and the washing buffer solution containing loosely bound aptamers was transferred into another scintillation cup. Cells were trypsinized, scraped off the plate, and transferred into a separate scintillation tube. Radioactivity was measured and quantified by a scintillation counter (LS 6500 Multipurpose Scintillation Counter; Beckmann). The percentage of bound aptamers was calculated by dividing the count rate of bound aptamers (cells) by the sum of bound (cells) and unbound (supernatant and wash buffers) count rates.

For the fluorescence-based binding assay, we employed aptamers labeled with red fluorescein (ATTO564) or green fluorescein (AlexaF488), which were chemically synthesized and purified by high-performance liquid chromatography (HPLC). Glass-bottom 24-well plates were used for cell plating. The aptamer concentration used for the binding experiments was 50 nM. To determine the binding constants, aptamer concentrations from 0 to 100 nM were employed with 2-fold serial dilutions. The volume of SBB buffer added in each well was 300 μL. Following the incubation of aptamers and the subsequent washing step (as described above), cells were fixed with 4% paraformaldehyde (PFA) for 10 min at room temperature and washed thrice with 1× PBS for binding and uptake experiments. For surface binding, cells were fixed prior to the incubation of aptamers. Fluorescence detection and quantification of bound aptamers were performed employing a plate reader (Infinite 200Pro; Tecan). The aptamer binding curve and Kd_app_ were analyzed employing SigmaPlot software (https://systatsoftware.com/products/sigmaplot/product-uses/sigmaplot-product-uses-analysis-of-ligand-binding-data/).

### Fluorescence In Situ Hybridization

Microscopy of surface-bound DNA aptamers was performed by confocal imaging (SP5; Leica). Stimulated RPTEC/TERT1 cells (CK^+^) were washed twice with 1× PBS, fixed with 4% PFA for 10 min at room temperature, and again washed thrice with 1× PBS. Subsequently, cells were incubated with 50 nM AlexaF488-labeled individual aptamers (Apta-1, Apta-4, and Apta-7) in SBB buffer for 30 min at 37°C. An Scr was used to determine the specificity of selected aptamers. Following incubation, cells were washed thrice with SELEX washing buffer and mounted on a glass slide employing Mowiol. Images were taken using the same confocal microscopy parameters for all of the samples.

For binding and uptake analysis, CK^+^ cells were incubated with 50 nM AlexaF488-labeled individual aptamers (Apta-1, Apta-4, Apta-7, and Scr) for 30 min at 37°C and were washed thrice with SELEX washing buffer. Cells were fixed, washed, and mounted on glass slides. Similar confocal microscopy parameters were employed for all of the images taken.

To investigate co-localization, CK^+^ and CK^−^ cells were treated with either 100 nM FITC-labeled Apta-7 or Scr as a control. Cells were washed twice with SELEX washing buffer, fixed with PFA, and washed thrice with 1× PBS. Subsequently, cells were permeabilized with 0.2% Triton X-100 for 5 min at room temperature and washed thrice with 1Mowiol PBS. Cells were blocked with 5% BSA for 30 min and incubated with anti-LAMP-1 mouse antibody in 3% BSA/1× PBS for 2 hr at room temperature. After incubation with the antibody, cells were washed thrice with 1× PBS, and the secondary antibody in 1% BSA/1× PBS was added to the cells. Following incubation for 1 hr at room temperature, cells were washed and incubated with DAPI for 10 min. Cells were again washed and mounted on a glass slide employing Mowiol.

### Cellular Uptake Inhibition Assay

Stimulated RPTEC/TERT1 (CK^+^) cells were either left untreated or treated with 200 μM chloroquine, a known inhibitor of clathrin-mediated endocytosis, for 2 hr at standard cell culture conditions. Subsequently, cells were washed with 1× PBS and incubated with 50 nM AlexaF488-labeled aptamers (Apta-1, Apta-4, Apta-7, and Scr) in SBB solution for 30 min at standard cell culture conditions. Following incubation, cells were washed twice with SELEX washing buffer, once with 0.5 M NaCl plus 0.2 N acetic acid to remove unbound or surface-bound aptamers, and again twice with 1× PBS. Cells were then fixed with 4% PFA for 10 min and were again washed thrice with 1× PBS. Cellular uptake of aptamers was analyzed and quantified by fluorescence detection employing a plate reader (Infinite 200Pro; Tecan).

### Statistical Analysis

The Student t test was used for statistical analysis.

## Author Contributions

G.R. designed and performed the experiments, analyzed the data, and wrote the manuscript; M.L. conducted the experiments and analyzed the data; H.S., Gert Mayer, and Günter Mayer contributed to manuscript writing and editing; A.P. established the protocol for the preliminary experiments; T.S. supervised experiments for the sucrose gradient centrifugation and participated in manuscript editing; and A.H. conceptualized and supervised the study and revised and approved the manuscript.

## Conflicts of Interest

The authors declare that they have no conflict of interest.

## Supplemental Information

Document S1. Figures S1–S4

Document S2. Article plus Supplemental Information

## References

[1] Kidney Disease: Improving Global Outcomes (2013). KDIGO 2012 clinical practice guideline for the evaluation and management of chronic kidney disease. Kidney Int. Suppl..

[2] Mills K.T., Xu Y., Zhang W., Bundy J.D., Chen C.S., Kelly T.N., Chen J., He J. (2015). A systematic analysis of worldwide population-based data on the global burden of chronic kidney disease in 2010. Kidney Int..

[3] Fox C.S., Matsushita K., Woodward M., Bilo H.J., Chalmers J., Heerspink H.J., Lee B.J., Perkins R.M., Rossing P., Sairenchi T., Chronic Kidney Disease Prognosis Consortium (2012). Associations of kidney disease measures with mortality and end-stage renal disease in individuals with and without diabetes: a meta-analysis. Lancet.

[4] Mahmoodi B.K., Matsushita K., Woodward M., Blankestijn P.J., Cirillo M., Ohkubo T., Rossing P., Sarnak M.J., Stengel B., Yamagishi K., Chronic Kidney Disease Prognosis Consortium (2012). Associations of kidney disease measures with mortality and end-stage renal disease in individuals with and without hypertension: a meta-analysis. Lancet.

[5] Vanholder R., Lameire N., Annemans L., Van Biesen W. (2016). Cost of renal replacement: how to help as many as possible while keeping expenses reasonable?. Nephrol. Dial. Transplant..

[6] Meng X.M., Nikolic-Paterson D.J., Lan H.Y. (2014). Inflammatory processes in renal fibrosis. Nat. Rev. Nephrol..

[7] Lee P.T., Chou K.J., Fang H.C. (2012). Are tubular cells not only victims but also perpetrators in renal fibrosis?. Kidney Int..

[8] Chevalier R.L. (2016). The proximal tubule is the primary target of injury and progression of kidney disease: role of the glomerulotubular junction. Am. J. Physiol. Renal Physiol..

[9] Risdon R.A., Sloper J.C., De Wardener H.E. (1968). Relationship between renal function and histological changes found in renal-biopsy specimens from patients with persistent glomerular nephritis. Lancet.

[10] Grgic I., Campanholle G., Bijol V., Wang C., Sabbisetti V.S., Ichimura T., Humphreys B.D., Bonventre J.V. (2012). Targeted proximal tubule injury triggers interstitial fibrosis and glomerulosclerosis. Kidney Int..

[11] Noone D., Licht C. (2014). Chronic kidney disease: a new look at pathogenetic mechanisms and treatment options. Pediatr. Nephrol..

[12] Wieser M., Stadler G., Jennings P., Streubel B., Pfaller W., Ambros P., Riedl C., Katinger H., Grillari J., Grillari-Voglauer R. (2008). hTERT alone immortalizes epithelial cells of renal proximal tubules without changing their functional characteristics. Am. J. Physiol. Renal Physiol..

[13] Slyne J., Slattery C., McMorrow T., Ryan M.P. (2015). New developments concerning the proximal tubule in diabetic nephropathy: in vitro models and mechanisms. Nephrol. Dial. Transplant..

[14] Sarközi R., Corazza U., Osterkamp J.P., Pirklbauer M., Mayer G., Schramek H. (2015). Synergistic induction of CCL2/MCP-1 expression driven by oncostatin M and IL-1β in human proximal tubular cells depends on STAT3 and p65 NFκB/RelA. Physiol. Rep..

[15] Sarközi R., Hauser C., Noppert S.J., Kronbichler A., Pirklbauer M., Haller V.M., Grillari J., Grillari-Voglauer R., Mayer G., Schramek H. (2011). Oncostatin M is a novel inhibitor of TGF-β1-induced matricellular protein expression. Am. J. Physiol. Renal Physiol..

[16] Dickinson H., Lukasser M., Mayer G., Hüttenhofer A. (2015). Cell-SELEX: in vitro selection of synthetic small specific ligands. Methods Mol. Biol..

[17] Ye M., Hu J., Peng M., Liu J., Liu J., Liu H., Zhao X., Tan W. (2012). Generating aptamers by cell-SELEX for applications in molecular medicine. Int. J. Mol. Sci..

[18] Tuerk C., Gold L. (1990). Systematic evolution of ligands by exponential enrichment: RNA ligands to bacteriophage T4 DNA polymerase. Science.

[19] Christensen E.I., Birn H., Storm T., Weyer K., Nielsen R. (2012). Endocytic receptors in the renal proximal tubule. Physiology (Bethesda).

[20] De S., Kuwahara S., Saito A. (2014). The endocytic receptor megalin and its associated proteins in proximal tubule epithelial cells. Membranes (Basel).

[21] Saito H., Takahashi S., Nagata M., Tsuchiya T., Mugishima H., Yan K., Kondo Y., Matsuyama T., Sekine T., Igarashi T. (2009). Reevaluation of glomerular charge selective protein-sieving function. Pediatr. Nephrol..

[22] de Araùjo M.E., Huber L.A., Stasyk T. (2008). Isolation of endocitic organelles by density gradient centrifugation. Methods Mol. Biol..

[23] Jones A.T. (2007). Macropinocytosis: searching for an endocytic identity and role in the uptake of cell penetrating peptides. J. Cell. Mol. Med..

[24] Mayor S., Pagano R.E. (2007). Pathways of clathrin-independent endocytosis. Nat. Rev. Mol. Cell Biol..

[25] Lodish H., Berk A., Zipursky S.L., Matsudaira P., Baltimore D., Darnell J. (2000). Receptor-mediated endocytosis and the sorting of internalized proteins. Molecular Cell Biology.

[26] Gorvin C.M., Wilmer M.J., Piret S.E., Harding B., van den Heuvel L.P., Wrong O., Jat P.S., Lippiat J.D., Levtchenko E.N., Thakker R.V. (2013). Receptor-mediated endocytosis and endosomal acidification is impaired in proximal tubule epithelial cells of Dent disease patients. Proc. Natl. Acad. Sci. USA.

[27] Goh L.K., Sorkin A. (2013). Endocytosis of receptor tyrosine kinases. Cold Spring Harb. Perspect. Biol..

[28] Huynh K.K., Eskelinen E.L., Scott C.C., Malevanets A., Saftig P., Grinstein S. (2007). LAMP proteins are required for fusion of lysosomes with phagosomes. EMBO J..

[29] Schneider T.D., Stephens R.M. (1990). Sequence logos: a new way to display consensus sequences. Nucleic Acids Res..

[30] Gellert M., Lipsett M.N., Davies D.R. (1962). Helix formation by guanylic acid. Proc. Natl. Acad. Sci. USA.

[31] Huppert J.L., Balasubramanian S. (2005). Prevalence of quadruplexes in the human genome. Nucleic Acids Res..

[32] Rhodes D., Lipps H.J. (2015). G-quadruplexes and their regulatory roles in biology. Nucleic Acids Res..

[33] Gatto B., Palumbo M., Sissi C. (2009). Nucleic acid aptamers based on the G-quadruplex structure: therapeutic and diagnostic potential. Curr. Med. Chem..

[34] Zuker M. (2003). Mfold web server for nucleic acid folding and hybridization prediction. Nucleic Acids Res..

[35] Fujita H., Imaizumi Y., Kasahara Y., Kitadume S., Ozaki H., Kuwahara M., Sugimoto N. (2013). Structural and affinity analyses of g-quadruplex DNA aptamers for camptothecin derivatives. Pharmaceuticals (Basel).

[36] Nagatoishi S., Isono N., Tsumoto K., Sugimoto N. (2011). Loop residues of thrombin-binding DNA aptamer impact G-quadruplex stability and thrombin binding. Biochimie.

[37] Schultze P., Macaya R.F., Feigon J. (1994). Three-dimensional solution structure of the thrombin-binding DNA aptamer d(GGTTGGTGTGGTTGG). J. Mol. Biol..

[38] Zhou J., Rossi J.J. (2011). Cell-specific aptamer-mediated targeted drug delivery. Oligonucleotides.

[39] Spurgeon K.R., Donohoe D.L., Basile D.P. (2005). Transforming growth factor-beta in acute renal failure: receptor expression, effects on proliferation, cellularity, and vascularization after recovery from injury. Am. J. Physiol. Renal Physiol..

[40] Han W.K., Bailly V., Abichandani R., Thadhani R., Bonventre J.V. (2002). Kidney injury molecule-1 (KIM-1): a novel biomarker for human renal proximal tubule injury. Kidney Int..

[41] Zhao X., Jiang C., Olufade R., Liu D., Emmett N. (2016). Kidney injury molecule-1 enhances endocytosis of albumin in renal proximal tubular cells. J. Cell. Physiol..

[42] Yang S.Y., Lin S.L., Chen Y.M., Wu V.C., Yang W.S., Wu K.D. (2016). Downregulation of angiotensin type 1 receptor and nuclear factor-κB by sirtuin 1 contributes to renoprotection in unilateral ureteral obstruction. Sci. Rep..

[43] Zhang G., Eddy A.A. (2008). Urokinase and its receptors in chronic kidney disease. Front. Biosci..

[44] Saito A., Sato H., Iino N., Takeda T. (2010). Molecular mechanisms of receptor-mediated endocytosis in the renal proximal tubular epithelium. J. Biomed. Biotechnol..

[45] Christensen E.I., Birn H. (2002). Megalin and cubilin: multifunctional endocytic receptors. Nat. Rev. Mol. Cell Biol..

[46] Schmid E.M., Ford M.G., Burtey A., Praefcke G.J., Peak-Chew S.Y., Mills I.G., Benmerah A., McMahon H.T. (2006). Role of the AP2 beta-appendage hub in recruiting partners for clathrin-coated vesicle assembly. PLoS Biol..

[47] Wang L.H., Rothberg K.G., Anderson R.G. (1993). Mis-assembly of clathrin lattices on endosomes reveals a regulatory switch for coated pit formation. J. Cell Biol..

[48] Daniels D.A., Chen H., Hicke B.J., Swiderek K.M., Gold L. (2003). A tenascin-C aptamer identified by tumor cell SELEX: systematic evolution of ligands by exponential enrichment. Proc. Natl. Acad. Sci. USA.

[49] Orend G., Chiquet-Ehrismann R. (2006). Tenascin-C induced signaling in cancer. Cancer Lett..

[50] Zueva E., Rubio L.I., Ducongé F., Tavitian B. (2011). Metastasis-focused cell-based SELEX generates aptamers inhibiting cell migration and invasion. Int. J. Cancer.

[51] Hanakahi L.A., Sun H., Maizels N. (1999). High affinity interactions of nucleolin with G-G-paired rDNA. J. Biol. Chem..

[52] Métifiot M., Amrane S., Litvak S., Andreola M.L. (2014). G-quadruplexes in viruses: function and potential therapeutic applications. Nucleic Acids Res..

[53] Yang D., Okamoto K. (2010). Structural insights into G-quadruplexes: towards new anticancer drugs. Future Med. Chem..

[54] Soundararajan S., Chen W., Spicer E.K., Courtenay-Luck N., Fernandes D.J. (2008). The nucleolin targeting aptamer AS1411 destabilizes Bcl-2 messenger RNA in human breast cancer cells. Cancer Res..

[55] Tang Z., Parekh P., Turner P., Moyer R.W., Tan W. (2009). Generating aptamers for recognition of virus-infected cells. Clin. Chem..

[56] Fleischhacker M., Schmidt B. (2007). Circulating nucleic acids (CNAs) and cancer--a survey. Biochim. Biophys. Acta.

[57] Siravegna G., Bardelli A. (2014). Genotyping cell-free tumor DNA in the blood to detect residual disease and drug resistance. Genome Biol..

[58] Oberbauer R., Schreiner G.F., Biber J., Murer H., Meyer T.W. (1996). In vivo suppression of the renal Na+/Pi cotransporter by antisense oligonucleotides. Proc. Natl. Acad. Sci. USA.

[59] Zinman B., Wanner C., Lachin J.M., Fitchett D., Bluhmki E., Hantel S., Mattheus M., Devins T., Johansen O.E., Woerle H.J., EMPA-REG OUTCOME Investigators (2015). Empagliflozin, cardiovascular outcomes, and mortality in type 2 diabetes. N. Engl. J. Med..

[60] Wanner C., Inzucchi S.E., Lachin J.M., Fitchett D., von Eynatten M., Mattheus M., Johansen O.E., Woerle H.J., Broedl U.C., Zinman B., EMPA-REG OUTCOME Investigators (2016). Empagliflozin and progression of kidney disease in type 2 diabetes. N. Engl. J. Med..

[61] DNASTAR. (2017). SeqMan NGen. http://www.dnastar.com/t-nextgen-seqman-ngen.aspx.

