# Supplementary material for: In Vitro Selection of Cell-Internalizing DNA Aptamers in a Model System of Inflammatory Kidney Disease
Source: Mol Ther Nucleic Acids. 2017 Jun 27;8:198–210. doi: 10.1016/j.omtn.2017.06.018 (PMC5504087; doi:10.1016/j.omtn.2017.06.018)
Supplement: Document S1. Figures S1–S4 [file mmc1.pdf]

OMTN, Volume *8*

Supplemental Information

In Vitro Selection of Cell-Internalizing DNA Aptamers in a Model System of Inflammatory Kidney Disease

Glory Ranches, Melanie Lukasser, Herbert Schramek, Andreas Ploner, Taras Stasyk, Gert Mayer, Günter Mayer, and Alexander Hüttenhofer

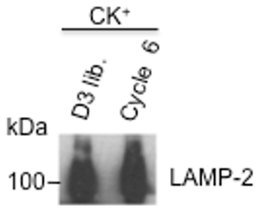

Figure S1. Western blot analysis of endosomal fractions containing the DNA aptamer pool from cytokine-stimulated cells. Endosomal lysates containing the pool of aptamers were analyzed employing an anti-LAMP-2 antibody which recognizes markers of late endosomes and lysosomes. The image is a representative blot of endosomal lysates from cytokine-stimulated (CK^+) cells, which were incubated either with the DNA aptamer library (D3 lib.) or the aptamer pool from cycle 6.

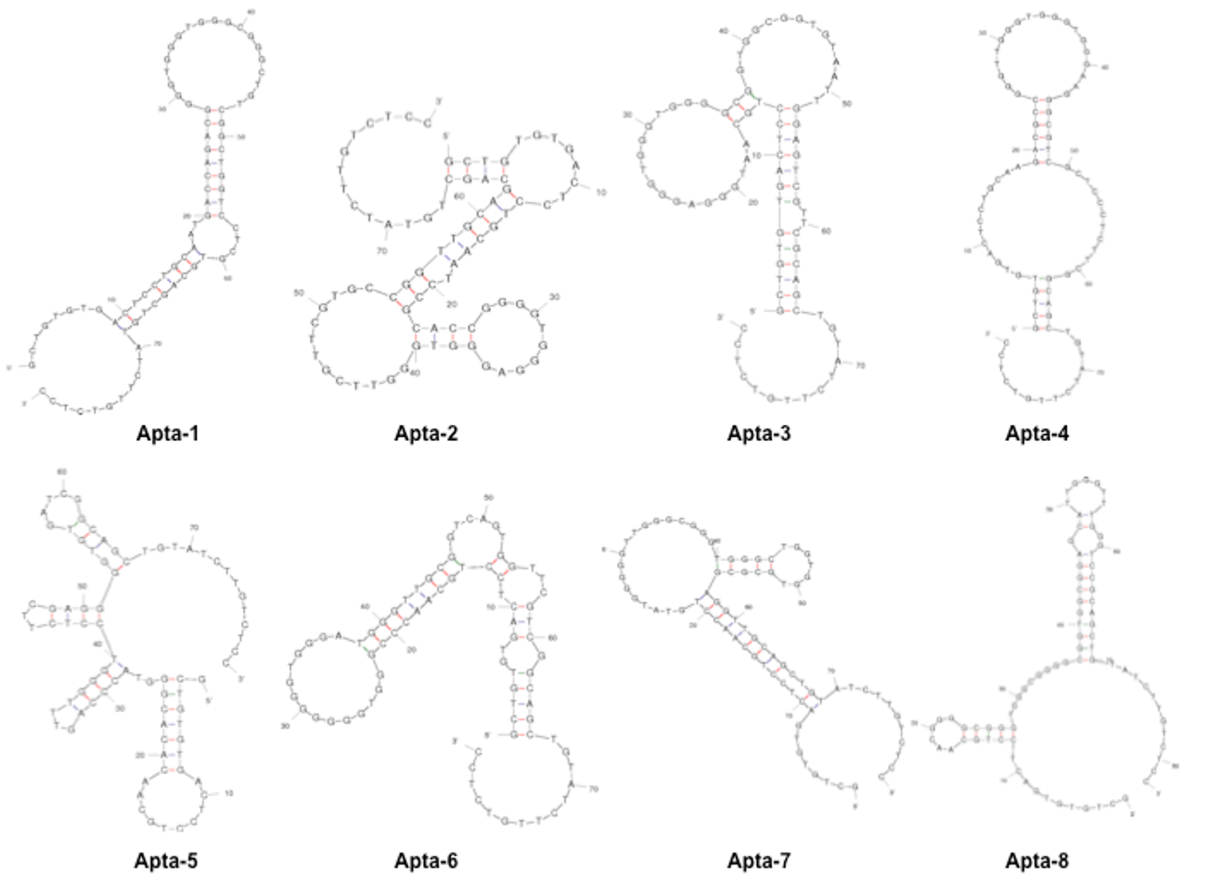

Figure S2. Predicted secondary structures of selected aptamers which target cytokine-stimulated cells. Sequences of selected aptamers were analyzed by employing the m-fold software. Primer sequences were included in the structure prediction since they might play a role in binding of target molecules located on cytokine-stimulated (CK^+) cells.

A

Aptamer ID	Sequences of full-length Apta-1 and its variants (5' to 3')
Apta-1	GCTGTGTGACTCCTGCAATGACCAGACGGGGTGGGTGGGCGGGCTGTCGGCTGGTCCTCGTGCAGCTGTATCTTGTCTCC
GQ-1	..............................TGACCAGACGGGGTGGGTGGGCGGGCTGTCGGCTGGTCCTCGT..............................
GQ-2	......................................CAGACGGGGGGGGTGGGCGGGCTGTCGGCTGG......................................

B

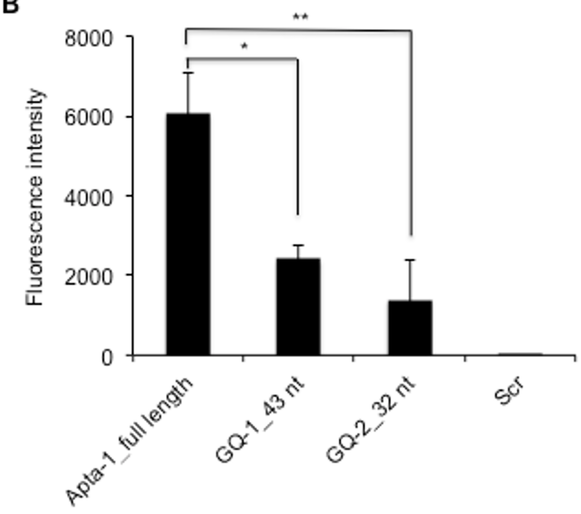

Figure S3. Binding of the full-length Apta-1 sequence and the corresponding minimal G-quartet sequence. A) The minimal Apta-1 binding motif was generated by removing the primer sequences (boxed), resulting to 43 nt randomized region (GQ-1), and the random sequences containing the G-quartet motif was further shortened to 32 nt (GQ-2). B) Cellular uptake of full-length Apta-1 and the corresponding minimal motif, and a scrambled (Scr) sequence was analyzed by fluorescence-based detection assay, employing CK^+ cells. The bar graph represents mean ± SD from two independent experiments. Student's t-test was performed for statistical analysis. * indicates $p<0.05$ and ** denotes $p<0.01$.

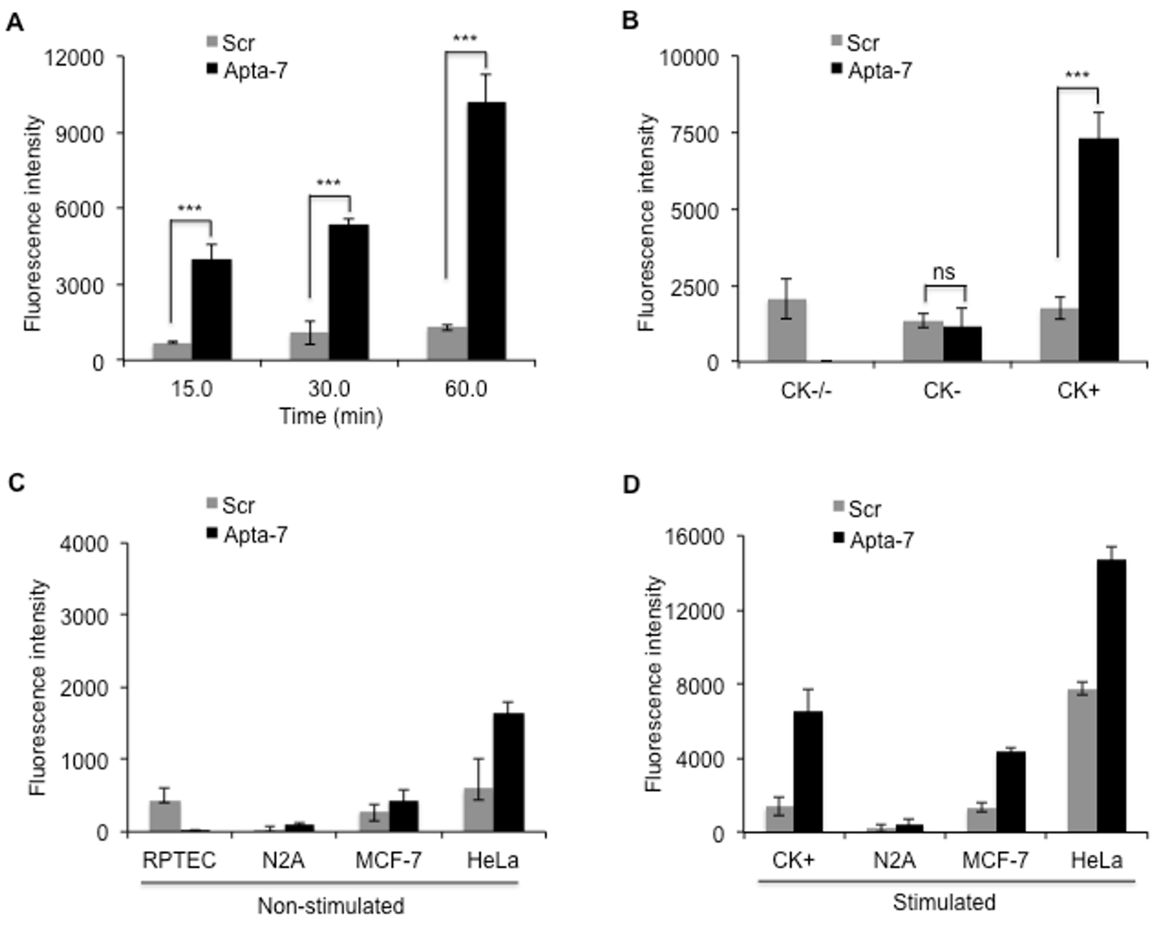

Figure S4. Specificity and time-dependent cellular uptake of DNA aptamer Apta-7. A) Cellular uptake of either ATTO-labeled Apta-7 or a scrambled (Scr) control sequence in cytokine-stimulated (CK^+) cells was analyzed in a time-dependent manner by a fluorescence-based detection assay employing a plate reader. Cells were incubated with 50 nM of aptamers at different time points: 0, 15, 30 and 60 min. Assays were performed in triplicates. The bar graph represents the mean±SD. Student's t-test was performed for statistical analysis. *** indicates $p<0.001$. B) Cellular uptake analysis of either the ATTO-labeled Apta-7 or a scrambled (Scr) sequence was performed in differentiated cells only ($CK^{-/-}$), supplement-starved and unstimulated (CK^-) cells, or supplement-starved and stimulated (CK^+) cells. Experiments were performed in triplicates. Student's t-test was performed for statistical analysis. The bar graph represents the mean ± SD. ns indicates non-significant and *** indicates $p<0.001$. Binding of Apta-7 to other cell lines under non-stimulated (C) or stimulated conditions (D), respectively. Cells were grown asynchronously, and were either untreated or treated with cytokines. ATTO-labeled Apta-7 or a scrambled (Scr) sequence was employed. The experiment was performed in six replicates. The bar graph represents the mean ± SD.
